# *ALK*基因状态与晚期肺腺癌患者一线培美曲塞化疗疗效的关系

**DOI:** 10.3779/j.issn.1009-3419.2017.11.02

**Published:** 2017-11-20

**Authors:** 梦阁 陈, 慧 曹, 瑛瑛 冀, 玉焕 毛, 淑景 申, 醒亚 李

**Affiliations:** 1 450052 郑州，郑州大学第一附属医院肿瘤科 Department of Medical Oncology, the First Affiliated Hospital of Zhengzhou University, Zhengzhou 450052, China; 2 450052 郑州，郑州大学第一附属医院放疗科 Department of Tumor Radiotherapy, the First Affiliated Hospital of Zhengzhou University, Zhengzhou 450052, China

**Keywords:** 肺肿瘤, ALK, 断裂融合基因, 培美曲塞, 一线化疗, Lung neoplasms, ALK, Fracture fusion gene, Pemetrexed, First-line chemotherapy

## Abstract

**背景与目的:**

间变性淋巴瘤激酶（anaplastic lymphoma kinase, *ALK*）是非小细胞肺癌（non-small cell lung cancer, NSCLC）的重要驱动基因之一，多项研究显示培美曲塞在ALK阳性肺癌中的疗效存在争议。本研究旨在继续探索以培美曲塞为基础的化疗在ALK阳性和阴性肺腺癌患者中的疗效。

**方法:**

回顾性分析郑州大学第一附属医院2015年1月-2016年4月经组织病理学证实的98例表皮生长因子受体（epidermal growth factor receptor, EGFR）、鼠类肉瘤病毒癌基因（kirsten rat sarcoma viral oncogene, *KRAS*）、鼠类肉瘤滤过性毒菌致癌同源体B1（V-rafmurine sarcoma viral oncogene homolog B1, BRAF）均为阴性的晚期肺腺癌患者的临床资料。分析*ALK*基因状态、临床特征、化疗疗效及无疾病进展生存期（progression-free survival, PFS）之间的关系。

**结果:**

98例患者均进行了*ALK*基因检测，*ALK*基因断裂融合34例（34.7%），未发生断裂融合64例（65.3%）。全部患者均接受一线培美曲塞联合铂类的化疗，客观缓解率（objective response rate, ORR）为21.4%，疾病控制率（disease control rate, DCR）为84.7%。ALK阳性肺腺癌患者的ORR和DCR均高于阴性患者（41.2% *vs* 10.9%, *χ*^2^=23.389, *P* < 0.001; 91.2% *vs* 81.3%, *χ*^2^=4.153, *P*=0.042），差异有统计学意义。*ALK*基因状态与年龄、性别、吸烟史、临床分期均无明显关系。ALK阳性肺腺癌的中位PFS为7.1个月（95%CI: 6.1-8.1），阴性4.7个月（95%CI: 3.818-5.582），二者的PFS差异有统计学意义（*χ*^2^=13.269, *P* < 0.001）。*Cox*回归多因素分析显示：培美曲塞联合铂类化疗的PFS与性别、年龄、吸烟、分期、与铂类药物的种类均无明显关系，ALK基因断裂融合是PFS相关的唯一变量（HR=0.392, 95%CI: 0.243-0.634, *P* < 0.001）。

**结论:**

ALK阳性相比ALK阴性肺腺癌患者一线应用以培美曲塞为基础的化疗有更大的临床获益。

## Abstract (en)

**Background and objective:**

Anaplastic lymphoma kinase (*ALK*) is one of the major driver genes of non-small cell lung cancer (NSCLC). Several studies have shown that the efficacy of pemetrexed in ALK-positive lung cancer is controversial. The aim of this study is to explore the efficacy of pemetrexed-based chemotherapy in patients with ALK-positive and negative lung adenocarcinoma.

**Methods:**

The clinical data of 98 cases of epidermal growth factor receptor (EGFR), kirsten rat sarcoma viral oncogene (*KRAS*), V-rafmurine sarcoma viral oncogene homolog B1 (BRAF)-negative patients with advanced lung adenocarcinoma patients who diagnosed by histopathology from January 2015 to April 2016 in the First Affiliated Hospital of Zhengzhou University were collected. The relationships between *ALK* gene status, clinical characteristics and response and progression-free survival (PFS) were analyzed.

**Results:**

All of the 98 patients' ALK status were determined. *ALK* gene fracture fusion occured in 34 cases (34.7%), no fracture fusion in 64 cases (65.3%). All patients underwent first-line pemetrexed and platinum-based chemotherapy, the objective response rate (ORR) was 21.4% and the disease control rate (DCR) was 84.7%. The ORR and DCR of patients with *ALK* fracture fusion were higher than those without fracture fusion (41.2% *vs* 10.9%, *χ*^2^=23.389, *P* < 0.001; 91.2% *vs* 81.3%, *χ*^2^=4.153, *P*=0.042), the difference was statistically significant. *ALK* gene status was not related to age, gender, smoking history and clinical stage. The median PFS of ALK-positive lung adenocarcinoma was 7.1 months (95%CI: 6.1-8.1) and negative was 4.7 months (95%CI: 3.818-5.582), and the difference was statistically significant (*χ*^2^=13.269, *P* < 0.001). *Cox* multivariate analysis indicates that PFS of pemetrexed combined with platinum chemotherapy was independent of gender, age, smoking, staging and platinum. *ALK* gene fracture fusion is an independent factor affecting PFS (HR=0.392, 95%CI: 0.243-0.634, *P* < 0.001).

**Conclusion:**

ALK-positive lung adenocarcinoma patients with first-line pemetrexed-based chemotherapy have greater clinical benefit than ALK-negative patients.

近年来随着空气污染的加重，肺癌发病率和死亡率仍然呈上升趋势。其中非小细胞肺癌（non-small cell lung cancer, NSCLC）占肺癌的70%-80%，其5年生存率仅为15%-17%^[1]^。有数据显示，约3/4的肺癌患者在发现时已是中晚期，失去最佳根治的时机。化疗仍然是治疗晚期肺腺癌患者不可或缺的方案。EML4-间变性淋巴瘤激酶（anaplastic lymphoma kinase, ALK）是在NSCLC患者肿瘤标本中由Soda等^[2]^于2007年首次发现，*EML4-ALK*融合基因是一种新兴的基因突变位点，它与肿瘤细胞的生长、增殖有密切关系^[3]^。国外Lee等^[4]^研究表明，ALK阳性肺腺癌患者培美曲塞化疗的疗效更佳。PROFILE 1014研究^[5]^显示，亚裔人培美曲塞联合顺铂或卡铂化疗，中位无疾病进展生存期（progression-free survival, PFS）为7.0个月，ORR为54%。PROFILE1029研究，即培美曲塞联合顺铂或卡铂一线治疗东亚人群ALK阳性晚期非鳞NSCLC患者Ⅲ期临床研究显示，中位PFS为6.8个月，客观缓解率（objective response rate, ORR）为46%。然而国外也有研究^[6]^报道ALK分型对于单独应用培美曲塞或非铂类药物及培美曲塞联合化疗方案的疗效及PFS均无影响，但是在不吸烟或少量吸烟的患者中，其中位生存期会有所增加。国内也有小样本研究指出，ALK分型对于化疗方案并无指导意义，即在铂类为基础的一线化疗方案中，不同方案的ORR与控制率相仿^[7]^。本研究的目的就在于探究在晚期肺腺癌患者中*ALK*基因状态和一线培美曲塞联合铂类化疗疗效及PFS之间的关系，为临床提供指导。

## 1 对象与方法

### 1.1 病例选择

入组标准：①肿瘤-淋巴结-转移（tumor-node-metastasis, TNM）分期标准采用国际肺癌研究协会（International Association for the Study of Lung Cancer, IASLC）2017年第七版分期标准，根据TNM分期证实的Ⅲ期和Ⅳ期表皮生长因子受体（epidermal growth factor receptor, EGFR）、鼠类肉瘤病毒癌基因（kirsten rat sarcoma viral oncogene, *KRAS*）、鼠类肉瘤滤过性毒菌致癌同源体B1（V-rafmurine sarcoma viral oncogene homolog B1, BRAF）均为阴性的NSCLC患者，初治选用培美曲塞联合铂类的一线双药化疗；②化疗期间未同步或序贯接受放疗或其他相关抗肿瘤治疗；③既往未接受过手术、放射治疗；④根据实体瘤疗效评价标准（Response Evaluation Criteria in Solid Tumors, RECIST）1.1^[8]^至少有1个可测量的病灶，定期复查计算机断层扫描（computed tomography, CT）评价疗效。

### 1.2 *ALK*融合基因的检测

*ALK*融合基因的检测方法包括增强免疫组化法（ventana-immunohistochemistry, V-IHC）、反转录聚合酶链反应法（reverse transcription-polymerase chain reaction, RT-PCR）和荧光原位杂交法（fluorescence *in situ* hybridization, FISH）。V-IHC使用的抗体为D5F3兔单克隆抗体；RT-PCR试剂盒为人类*EML4-ALK*融合基因检测试剂盒，通过多重引物RT-PCR法，需0.1 μg-0.5 μg肿瘤组织的RNA，即可检测出*EML4-ALK*融合基因的阳性信号；FISH检测使用的试剂盒为vysis ALK-FISH-break-apart Kit，*ALK*断裂融合的肿瘤细胞特征是橘红色和绿色信号互相分离，间距至少超过2个信号直径，无*ALK*断裂融合肿瘤细胞特征是橘红色和绿色信号重合为黄色或者相互粘合。

### 1.3 疗效评价与随访

治疗效果评价标准按RECIST标准判定，分为完全缓解（complete response, CR）、部分缓解（partial response, PR）、稳定（stable disease, SD）以及病情进展（progressive disease, PD），其中ORR＝（完全缓解+部分缓解）/全部病例×100%^[9]^，计算ORR（即CR+PR所占比例）、疾病控制率（disease control rate, DCR）（即CR+PR+SD所占比例）。主要研究终点为PFS，PFS定义为第1次用化疗药的时间至首次记录的PD时间或死亡时间。随访截止至2017年10月。

### 1.4 统计学方法

采用SPSS 17.0统计学软件进行数据的统计学处理。分析*ALK*基因状态、临床特征与化疗疗效之间的关系采用卡方检验或*Fisher’s*确切概率法。生存分析采用*Kaplan-Meier*法并进行*Log-rank*检验。*Cox*回归进行PFS的多因素分析。所有统计检验均为双侧概率检验，以*P* < 0.05为差异有统计学意义。

## 2 结果

### 2.1 一般资料

回顾性分析郑州大学第一附属医院2015年1月-2016年4月经组织病理学证实的107例EGFR、KRAS、BRAF均为阴性的晚期肺腺癌患者的临床资料。所有患者均进行了*EGFR*、*KRAS*、*BRAF*、*ALK*基因检测。培美曲塞均为国产用药，联合铂类化疗4周-6周期后，若无疾病进展，采用培美曲塞维持治疗。所有患者均接受叶酸、维生素B_12_补充剂和地塞米松，以避免药物不良反应。采用电话随访，随访至2017年10月，随访时间2.0个月-22.4个月，中位随访时间7.8个月。排除随访丢失的患者，98例患者纳入此研究。其中男性57例，女性41例；年龄25岁-75岁，中位年龄为57岁；一线化疗中位化疗周期数为6个（2-14）；其中58例进行了培美曲塞维持治疗，维持周期数为2个-10个；Ⅳ期患者79例（80.6%），Ⅲb期17例（17.3%），Ⅲa期2例（2.1%）。

### 2.2 *ALK*基因状态与临床病理特征之间的关系

85例患者由V-IHC检测，10例FISH，3例RT-PCR。其中RT-PCR法基因检测有1例未发生*ALK*融合，1例发生*EML4-ALK*融合，1例同时发生*EML4-ALK*及*LOC388942-ALK*基因融合。*ALK*阳性肺腺癌患者34例（34.7%），ALK阴性肺腺癌为64例（65.3%）。*ALK*基因断裂融合与年龄、性别、吸烟史、临床分期均无关（表 1）。

**1:** *ALK*基因状态与临床病理特征的关系 Relationships between ALK status and clinicopathologic charecteristics

Characteristic	ALK positive	ALK negative	*χ*^2^	*P*
Gender			0.583	0.445
Male	18 (52.9%)	39 (60.9%)		
Female	16 (47.1%)	25 (39.1%)		
Age (yr)			0.983	0.322
≥65	6 (17.6%)	17 (26.6%)		
< 65	28 (82.4%)	47 (73.4%)		
TNM stage			3.354	0.187
Ⅲa	1 (2.9%)	1 (1.6%)		
Ⅲb	9 (26.5%)	8 (12.5%)		
Ⅳ	24 (70.6%)	55 (85.9%)		
Smoking history			2.108	0.147
No	28 (82.4%)	44 (68.8%)		
Yes	6 (17.6%)	20 (31.2%)		
TNM: tumor-node-metastasis.				

### 2.3 *ALK*基因状态与化疗疗效之间的关系

98例患者均有可评价病灶。其中CR 0例（0%），PR 21例（21.4%），SD 62例（63.3%），PD 15例（15.3%），ORR为21.4%，DCR为84.7%。疗效评价见表 2。ALK阳性肺腺癌患者的ORR和DCR均高于阴性患者（41.2% *vs* 10.9%, *χ*^2^=23.389, *P* < 0.001; 91.2% *vs* 81.3%, *χ*^2^=4.153, *P*=0.042），差异有统计学意义。

**2:** *ALK*基因状态与化疗疗效 Response to treatment according to ALK status

Efficacy evaluation	ALK positive	ALK negative	*χ*^2^	*P*
CR	0	0		
PR	14 (41.2%)	7 (10.9%)		
SD	17 (50.0%)	45 (70.3%)		
PD	3 (8.8%)	12 (18.6%)		
ORR	41.2%	10.9%	23.389	< 0.001
DCR	91.2%	81.3%	4.153	0.042
CR: response rate; PR: partial response; SD: stable disease; PD: progressive disease; ORR: objective response rate; DCR: disease control rate.				

### 2.4 *ALK*基因状态与PFS之间的关系

单因素分析显示，ALK阳性肺腺癌的中位PFS为7.1个月（95%CI: 6.1-8.1），阴性为4.7个月（95%CI: 3.818-5.582），二者PFS差异有统计学意义（*χ*^2^=13.269, *P* < 0.001）（图 1）。*Cox*回归多因素分析显示：培美曲塞联合铂类化疗的PFS与性别、年龄、吸烟、分期、与铂类药物的种类均无关，*ALK*基因断裂融合是PFS相关的唯一变量（HR=0.392, 95%CI: 0.243-0.634, *P* < 0.001）。

**1:**
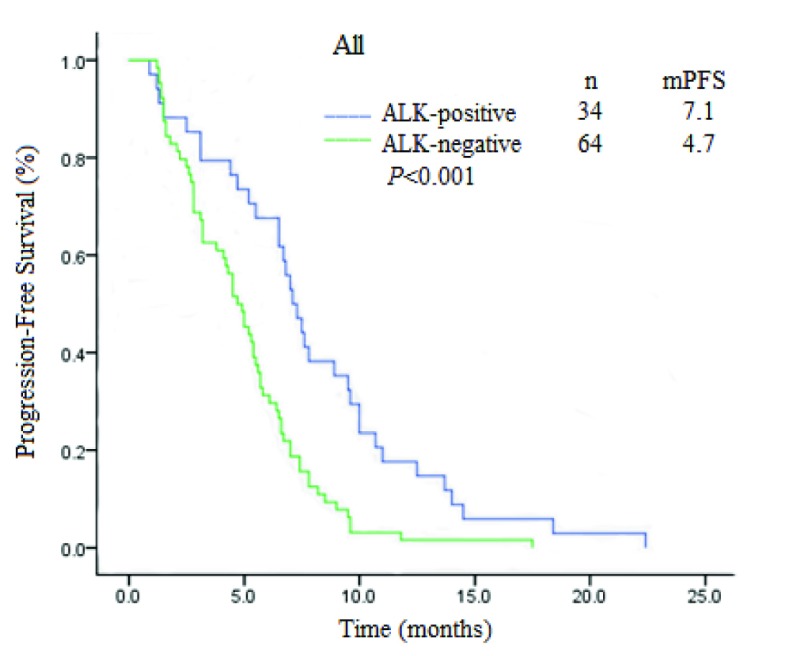
ALK阳性与ALK阴性肺腺癌患者PFS生存曲线比较 Comparison of PFS in ALK-positive with ALK-negative lung adenocarcinoma patients. PFS: progression-free survival.

## 3 讨论

靶向治疗在基因融合患者中的功效已得到证实，尽管这些融合患者化疗的功效仍然存在争议。目前克唑替尼在2011年获得美国药品食品管理局（Food and Drug Administration, FDA）批准。这是美国国立综合癌症网络（National Comprehensive Cancer Network, NCCN）提出的ALK阳性肺癌患者一线治疗的首选药物^[10]^。与化疗相比，克唑替尼用于初治的ALK阳性晚期NSCLC患者，其ORR和PFS均有显著提高^[5]^，用于经治的ALK阳性晚期NSCLC患者的临床疗效也明显优于培美曲塞或多西他赛化疗^[11]^。但是，由于这种药物尚未进入医保，许多ALK阳性患者无法承担费用。在临床研究中发现晚期肺腺癌患者接受一线克唑替尼靶向治疗的极少，大多数以化疗为首选，常用的化疗方案为铂类联合培美曲塞、紫杉醇、多西他赛、吉西他滨等药物。

培美曲塞是一种有效的胸苷酸合酶（thymidylate synthase, TS）抑制剂和其他参与嘌呤和嘧啶合成的叶酸依赖性辅酶抑制剂。有研究^[12, 13]^探讨了ALK阳性NSCLC组织TS的表达水平，发现ALK阳性患者TS蛋白处于低表达状态。随机试验^[14, 15]^表明，TS阴性NSCLC患者比阳性患者更能从以培美曲塞为基础的化疗中获益，这可能部分解释了*ALK*断裂融合基因能增加培美曲塞铂二联的疗效。

目前推荐的*ALK*基因检测的方法有V-IHC、RT-PCR和FISH。已有研究^[16, 17]^发现应用V-IHC法对*ALK*断裂融合基因进行检测的结果，经FISH和RT-PCR法验证后，其结果具有高度的一致性。

*EML4-ALK*基因阳性肺癌是NSCLC的重要亚型，约占NSCLC的5%^[18, 19]^，而在*EGFR*、*KRAS*、*HER2*或*TP53*等基因未发生突变的NSCLC患者中，*EML4-ALK*融合阳性的比例达25%^[20, 21]^，部分报道中EGFR、KRAS均为野生型的腺癌中EML4-ALK阳性比例高达30%-42%^[22, 23]^。本研究中，*ALK*基因断裂融合占肺腺癌的34.7%，与国外部分报道相近但略高。在本研究中培美曲塞联合铂类化疗的PFS与含铂种类无关，然而国内有研究^[24]^显示晚期肺腺癌患者一线培美曲塞联合奈达铂优于顺铂。分析这些差异可能与种族地域不同、样本量小、选择偏倚、病理类型等因素有关。

目前国内有关*ALK*基因状态与一线培美曲塞联合铂类化疗疗效之间关系的报道较少。有研究^[8]^报道培美曲塞联合铂类一线化疗中，ALK阳性组与ALK阴性组有效率和控制率无统计学差异。国外Park等^[25]^报道52例ALK阳性组的反应率为26.9%，中位PFS为7.8个月，均高于ALK阴性组。在本研究中，ALK阳性组ORR为41.2%，中位PFS为7.1个月，这与PROFILE 1014研究^[5]^以及PROFILE 1029研究结果也类似。表明ALK阳性肺腺癌患者对一线培美曲塞化疗更有效，*ALK*基因状态可能能够预测培美曲塞的益处，对化疗方案有指导意义。这项回顾性研究不如前瞻性研究那么强大。与任何回顾性分析一样，我们的研究有固有的限制。主要的限制之一是研究人群，特别是ALK阳性队列，可能有受到抽样偏差影响。在本研究中，ALK阳性肺腺癌患者仅为34例，不排除选择偏倚和样本量小等因素的影响，有待大样本、多中心、前瞻性研究进一步证实。

综上所述，*ALK*基因是晚期肺腺癌患者一线培美曲塞化疗PFS的预测因素，相比ALK阴性肺腺癌患者，ALK阳性患者一线应用以培美曲塞为基础的化疗有更大的临床获益。
